# Therapeutics and Materia Medica—Borate of Quinine; Tannate of Cannabin; Antipyrine; Salicylic Acid in the Treatment of Cerebro-spinal Meningitis; Some Therapeutic Uses for Lobelia; Asclepias Incarnata

**Published:** 1884-11-20

**Authors:** 


### THERAPTTICS AND MATERIA MEDICA.

### BORATE OF QUININE.

Among the new salts of quinine are some that deserve a trial, being cheaper than the officinal salts and more easy of administration. The borate is an amorphous, yellowish powder, of a mild, not very bitter taste. It is much more soluble than the sulphate, dissolving, it is said, in an equal quantity of water. In some experiments conducted by Drs. Finkler and Prior, of Bonn (Deutsches Med. Wochenschrift), it was found that it agreed very well with irritable stomachs, and produced no unfavorable effects upon digestion, even after several weeks' administration. It had the decided antithermic powers of the officinal salts, but caused less nervous depression, and tinnitus aurium was not noticed. It was especially recommended that when given in large doses (varying from eight to sixteen grains every hour or half-hour until forty-eight grains were swallowed) it should be well diluted, and accompanied by a little brandy. Borate of quinine seems especially adapted for treatment of children's diseases, as it is very soluble, and, on account of its mild taste, is easily administered.

### TANNATE OF CANNABIN.

Experience has served to support still further the claims of this drug as a pure hypnotic. Dr. Trohmuller (Allgem. Med.-Chir. Zeitung) reports excellent results from its use in insomnia of phthisis and various other diseases. He considers it a powerful rival to opium (in doses of one to five grains), as it has no effect upon the secretions and is less likely to cause toxic effects.

### ANTIPYRINE.

Favorable notices have appeared lately in the journals upon the derivative of chinoline, known as antipyrine, discovered by Dr. Knorr,* of Erlangen, who has kept its method of manufacture a secret for the present and patented it, but promises a future communication on the subject. It is said, also, to be made synthetically by the reaction of acetic ether on aniline.

The drug is in the form of a white crystalline powder, very soluble in water (one to three), with a feeble odor, and a slightly bitter taste easily covered by some aromatic water or wine. Professor Filehne, having given the new remedy a trial in various febrile affections, acute and chronic, found that by doses of five or six grammes a high temperature was quickly reduced to the normal.

* Zeitschrift fur Klinische Medizin, Bd. vii. Heft 6.

These doses were generally well borne, but occasionally vomiting occurred, the precaution being taken to give the drug in 10-gramme doses each hour until the desired amount had been administered. The duration of the effect is variable, but usually the temperature does not again increase until seven or nine hours afterwards, and sometimes the result is prolonged for eighteen or twenty hours. The temperature commences to fall within half an hour after the first dose, and its maximum effect is attained from three to five hours afterwards. In children, debilitated patients, and phthisical cases it is advised to give smaller doses. The frequency of the pulse decreases at the same time with the temperature, but without following any rigid proportion. No albumen has been found in the urine, nor has this fluid shown any marked change of color under its use. Respiration also appears to be uninfluenced by it.

Subsequent experiments made by Drs. May, of Cologne, and Rank, of Stuttgart, have confirmed the preceding observations as to the antipyretic action of the new drug. In order to avoid vomiting, which occasionally will occur, Dr. Rank has tried hypodermic injections of the drug : the solubility in cold water (one to three) requiring too large doses for hypodermic injections, he used warm water, fifty centigrammes of which will dissolve one gramme without precipitation upon cooling, the solution remaining for a week perfectly limpid. Used in this way, antipyrine no longer caused vomiting, while at the same time producing the usual effect upon the temperature. In not a single case was there any accident to report, not the least inflammatory reaction, and no abscesses.

Dr. Rapin reports,* however, that Erb tried the hypodermic method, but gave it up on account of the severe pain caused by these injections ; he did this the more willingly because the remedy rarely disagrees with the stomach.

From the results obtained in Erb's cases, it was concluded by Dr. Rapin† that antipyrine is superior in its antipyretic action to all the agents ordinarily given to reduce combustion, such as quinine, salicylic acid, kairine, etc. ; the results obtained from its use more complete, and the absence of all evil effects after its ingestion constitutes an additional advantage. Finally, its price is much lower than quinine ; but this advantage is merely a nominal one at present, because of the larger quantity required to produce the same effect.

The mode of action of antipyrine is in all probability very analogous to that of kairine, which has been found by Mr. Loyd to reduce greatly the amount of gases taken up by the blood, and particularly of hydrogen, the spectroscope showing attenuation and disappearance of the bands of oxyhæmoglobin. Should this prove to be true as applied to antipyrine, it would tend to impair its value in typhoid and other low fevers which tend in a marked degree to destroy the blood-corpuscles. M. Quinquand‡ found that the amount of gas in the blood is not materially affected by small doses of kairine, but it is by large doses. Since these remedies possess

---

* Revue Medicale de la Suisse Romande, No. 7, 1884.

† Loc. cit.

‡ Progres Medical, May 10, 1884.

some of the properties of quinine, it is possible that they may also exert a toxic action upon the heart, which might render them dangerous in cases of depression of the vital powers, when given in large doses.

### SALICYLIC ACID IN THE TREATMENT OF CEREBRO-SPINAL MENINGETIS.

Having been impressed, during an epidemic of cerebro-spinal meningetis, with the idea that an analogy existed between the disease and acute rheumatism, Dr. Ramsey* was led to use salicylic acid in its treatment. During the epidemic mentioned, eleven cases of cerebro-meningetis occurred in his practice. Four of them were treated with ergot, potassium bromide, chloral, aconite, quinine sulphate, morphine sulphate, and numerous other remedies : the result was three deaths and one recovery. The seven other cases were treated with saliclylic acid, with the result of five recoveries and but two deaths. They were all violent cases, and possessed the distressing characteristics of the disease in full. He says that salicylic acid will control the migratory pains of head, elbow, and knee, and reduce the temperature, in most, if not all, cases of cerebro-spinal meningetis, and that in every case in which he prescribed it great beneft in the condition of the patients from the beginning of its use.

### SOME THERPEUTIC USES OF LOBELIA.

In a recent communication to the Societie de Therapeutique. Dr. Fournier† states that he has successfdlly used the tincture of *Lobelia inflata*, in doses of *m*xv–xxx, in several cases of cardiac dyspnœa, and with good results in the third stage of phthisis. As lobelia alone is nauseating, it should be combined with polygala. Huchard uses the following formula, which is well borne by his patients :

℞ Potassi iodidi.......................................ʒ ij

Tinct. lobeliæ..} aa..............................f ʒ ij

Tinct. polygalæ}

Ext. opii.............................................gr. ss

Aquam destillatum ad..........................f ℥ viij.

M. Dose, a tablespoonful morning and evening in chronic bronchitis and asthma.

M. C. Paul has used lobelia and iodide of potassium with success in catarrhal asthma, giving *m*xx of tincture of lobelia to gr. viii of potassium.

### ASCLEPIAS INCARNATA.

Dr. J. Hosack Fraser,‡ after three months' clinical trial of the American diuretic drug, *Asclepias incarnata*, or white Indian hemp, thus summarizes its physiological actions :

1. *On the Circulatory System.*—It stiulates the cardiac contract-

---

* Western Medical Reporter, March, 1884.

† ‡ Practioner.

ions and increases the blood-pressure. For two hours after the administration of forty minims of the liquid extract, there is an increase in the strength and volume of the pulse. It steadies the cardiac contractions, and assists in restoring the rhythm of the pulse when any irregularity exists. The stage of stimulation is sometimes followed by slight depression of the circulation, and in order to maintain its stimulating effects, it should be repeated at intervals of three hours.

2. *On the Urinary System.*—It is a speedy, potent, and reliable diuretic. He has found it to succeed admirably in cases where the best known diuretics had entirely and signally failed. It is invaluable in all forms of dropsy, but especially so in dropsy of cardiac and renal origin. He believes that it acts on the kidneys in precisely the same manner as digitalis. One great advantage which it possesses is that it does not cause any gastro-intestinal disturbance ; on the contrary, it frequently acts as a stomachic tonic.

The root is the part which is used in medicine, and is said to possess all the medical properties of the plant. It may be administered in the form either of an infusion or of a fluid extract. The latter preparation should be given in ʒss to ʒi doses every three hours. Smaller doses are useless.—*Medical Times.*

